# Editorial: Advanced approaches in pediatric clinical pharmacology

**DOI:** 10.3389/fphar.2024.1372290

**Published:** 2024-01-30

**Authors:** Hesham Al-Sallami, Andrea Diniz, Jaydeep Sinha, Eleni Karatza, Karel Allegaert

**Affiliations:** ^1^ School of Pharmacy, University of Otago, Dunedin, New Zealand; ^2^ Laboratory of Pharmacokinetics and Biopharmacy, Department of Phamacy, Universidade Estadual de Maringá, Maringá, Paraná, Brazil; ^3^ Department of Pediatrics, UNC School of Medicine, The University of North Carolina at Chapel Hill, Chapel Hill, NC, United States; ^4^ Division of Pharmacotherapy and Experimental Therapeutics, UNC Eshelman School of Pharmacy, The University of North Carolina at Chapel Hill, Chapel Hill, NC, United States; ^5^ Department of Development and Regeneration, KU Leuven, Leuven, Belgium; ^6^ Department of Pharmaceutical and Pharmacological Sciences, KU Leuven, Leuven, Belgium; ^7^ Department of Hospital Pharmacy, Erasmus MC, Rotterdam, Netherlands

**Keywords:** pediatric clinical pharmacology, pediatric pharmacotherapy, pharmacokinetics, efficacy, safety, pharmacodynamics

Clinical pharmacology focuses on the impact of intrinsic and extrinsic factors on the inter-and intra-subject variability in drug exposure (pharmacokinetics) and response (pharmacodynamics, covering effects and side-effects). The aim hereby is to provide the right patient the right drug, using the right dose and at the right time. Traditional clinical pharmacology methods involve primarily performing large (randomized, placebo-controlled) trials to inform dosing strategies, including potential dose adjustments based on patients characteristics. Such studies are commonly conducted in homogeneous patient cohorts of adults. However, ethical challenges like the use of placebo or a control arm in an off-label pharmacotherapy setting, as well as logistical challenges like adequate number of study participants, and the fact that children are rather heterogeneous on age and weight are additional burdens in pediatric clinical pharmacology. Consequently, advanced approaches and additional creativity are needed to make pediatric drug development plans impactful on pediatric pharmacotherapy.

Advanced approaches hereby cover pharmacometrics, clinical decision support tools, different approaches related to allometric scaling, real world data, preclinical findings (*in vitro* studies, juvenile animal models), as well as biomarker development adapted to children (Van den Anker et al., 2018; Smits et al., 2022). The key characteristic of pediatrics is biological maturation, resulting in age-dependent physiology, potentially affecting pharmacology. Biological maturation involves growth (co-linear with weight), differentiation and development, which extends within pediatrics from preterm neonates and infants through children to adolescents and young adults. To further stress the heterogenous character of this population, there is a >100-fold variability in weight (<0.5 to >50 kg). These age-dependent physiological changes are further modulated by non-maturational covariates like disease, pharmacogenetics or drug-drug interactions (Allegaert et al., 2017; Smits et al., 2022).

Although the scope of this Research Topic was broad, the majority of papers addressed pharmacotherapy of the central nervous system, with focus on preoperative sedation and anesthesia induction in children undergoing surgery (oral midazolam to intranasal dexmedetomidine) (Cai et al.), on the safety and efficacy of remimazolam to induce and maintain general anesthesia during elective surgery in children (Fang et al.), and on the effectiveness and safety of perampanel versus oxcarbazepine as monotherapy in children and adolescents with newly diagnosed focal epilepsy (Yi et al. and Yi et al.). Finally, this Research Topic also reports on a comparative randomized clinical trial on the efficacy and safety of tacrolimus versus hydrocortisone as topical treatment of atopic dermatitis in children (Mohamed et al.).

The research community and readers should realize that the developmental physiology of the central nervous system occurs throughout pediatric life up to young adulthood, as this includes brain growth, the ontogeny of receptors, and development of the connectome and functional connectivity. These maturational changes in receptor expression and function result in age-specific sensitivity to develop epilepsy (Rakhade and Jensen, 2009). Structural networks develop earlier than functional networks, while the functional networks demonstrate more dramatic maturational changes with the evolution of structural networks serving as the anatomical backbone (Cao et al., 2016). Liu et al. illustrated the relevance to pediatric pharmacotherapy when describing significant differences between adolescent and adult safety profiles of antipsychotic and antidepressant drugs, with risk differences ranging from 9.6% to 36.6%, most pronounced for sedation as being more commonly reported in adolescents (Liu et al., 2019).

When assessing the study to compare preoperative sedation and anesthesia induction in children undergoing surgery (oral midazolam to intranasal dexmedetomidine) (Cai et al.), some typical pediatric aspects emerged. This included the relevant age range (2–6 years), when the need of premedication as part of integrated peri-anesthesia care is highest. Furthermore, the primary and secondary outcomes are also well tailored to children like mask acceptance for subsequent inhalation induction, and behavioral scores, parental separation anxiety scores, and the postoperative incidence of emergence agitation and recovery time. This reflects the creativity needed to design a study that is both valid (validated biomarkers for this age group) and patient-relevant (including parents). In the remimazolam to propofol protocol, a similar age range (3–6 years) is targeted, age-appropriate scores (sedation = Modified Observer’s Assessment of Alertness/Sedation scale, pain = Face, Legs, Activity, Cry, Consolability; Post-Hospitalization Behavior Questionnaire for Ambulatory Surgery), tools (Bispectral Index values over time) and outcome (parental satisfaction, emergence delirium) are collected as secondary outcome variables (Fang et al.). Finally, focused on newly diagnosed focal epilepsy (4–18 years), the effectiveness and safety of perampanel was compared to oxcarbazepine (Yi et al. and Yi et al.). Primary outcome was seizure freedom rate at 6 months, with retention rates up to 12 months as secondary outcomes. While there was no difference in effectiveness between both treatment strategies, the authors also reported in detail on the tolerability and safety of both treatment regimes, so that this information is supportive to guide practices and to improve the quality of shared decision processes.

A comparative randomized clinical trial evaluating the efficacy and safety of topical tacrolimus or hydrocortisone to treat atopic dermatitis in children (2–16 years, 200 cases) further completed the Research Topic (Mohamed et al.). An emollient (Vaseline petrolatum jelly) was hereby co-applied in both groups, as this is commonly used. This improves the clinical relevance of any additional pharmacotherapeutic effect. Clinical assessment was based on a validated score (modified Eczema Area and Severity Index score). Besides surface, morphology, and severity of lesions, pruritus is also included in this score. This symptom has recently been highlighted as the most relevant patient-outcome in children with atopic dermatitis. While clinical researchers commonly focus on skin assessment, children value reduction or elimination of pruritus. Secondary outcomes were related to modulation of the systemic inflammation, or treatment-related toxicity.

In conclusion, this Research Topic on advanced approaches in pediatric clinical pharmacology mainly illustrates the complexity and creativity needed to facilitate clinical trial conduct, crucial to generate data to support pediatric pharmacotherapy.

## References

[B1] Allegaert K. Simons S. H. P. Tibboel D. Krekels E. H. Knibbe C. A. van den Anker J. N. (2017). Non-maturational covariates for dynamic systems pharmacology models in neonates, infants, and children: filling the gaps beyond developmental pharmacology. Eur. J. Pharm. Sci. 109S, S27-S31–S31. 10.1016/j.ejps.2017.05.023 28506866

[B2] Cao M. Huang H. Peng Y. Dong Q. He Y. (2016). Toward developmental connectomics of the human brain. Front. Neuroanat. 10, 25. 10.3389/fnana.2016.00025 27064378 PMC4814555

[B3] Liu X. I. Schuette P. Burckart G. J. Green D. J. La J. Burnharm J. M. (2019). A comparison of pediatric and adult safety studies for antipsychotic and antidepressant drugs submitted to the United States Food and Drug Administration. J. Pediatr. 208, 236–242. 10.1016/j.jpeds.2018.12.033 30679050 PMC7171692

[B4] Rakhade S. N. Jensen F. E. (2009). Epileptogenesis in the immature brain: emerging mechanisms. Nat. Rev. Neurol. 5 (7), 380–391. 10.1038/nrneurol.2009.80 19578345 PMC2822660

[B5] Smits A. Annaert P. Cavallaro G. De Cock P. A. J. G. de Wildt S. N. Kindblom J. M. (2022). Current knowledge, challenges and innovations in developmental pharmacology: a combined conect4children expert group and European Society for Developmental, Perinatal and Paediatric Pharmacology white paper. Br. J. Clin. Pharmacol. 88 (12), 4965–4984. 10.1111/bcp.14958 34180088 PMC9787161

[B6] Van den Anker J. Reed M. D. Allegaert K. Kearns G. L. (2018). Developmental changes in pharmacokinetics and pharmacodynamics. J. Clin. Pharmacol. 58 (Suppl. 10), S10-S25–S25. 10.1002/jcph.1284 30248190

